# Supplementary material for: Splice-Junction-Based Mapping of Alternative Isoforms in the Human Proteome
Source: Cell Rep. Author manuscript; Available in PMC 2020 Jan 15. (PMC6961840; doi:10.1016/j.celrep.2019.11.026)
Supplement: 2 [file NIHMS1546469-supplement-2.zip › DF1/PXD009737/TestisTrypsin/TestisTrypsin_45_REEP1_GTAVVLIFGTLYPAYYSYK.pdf]

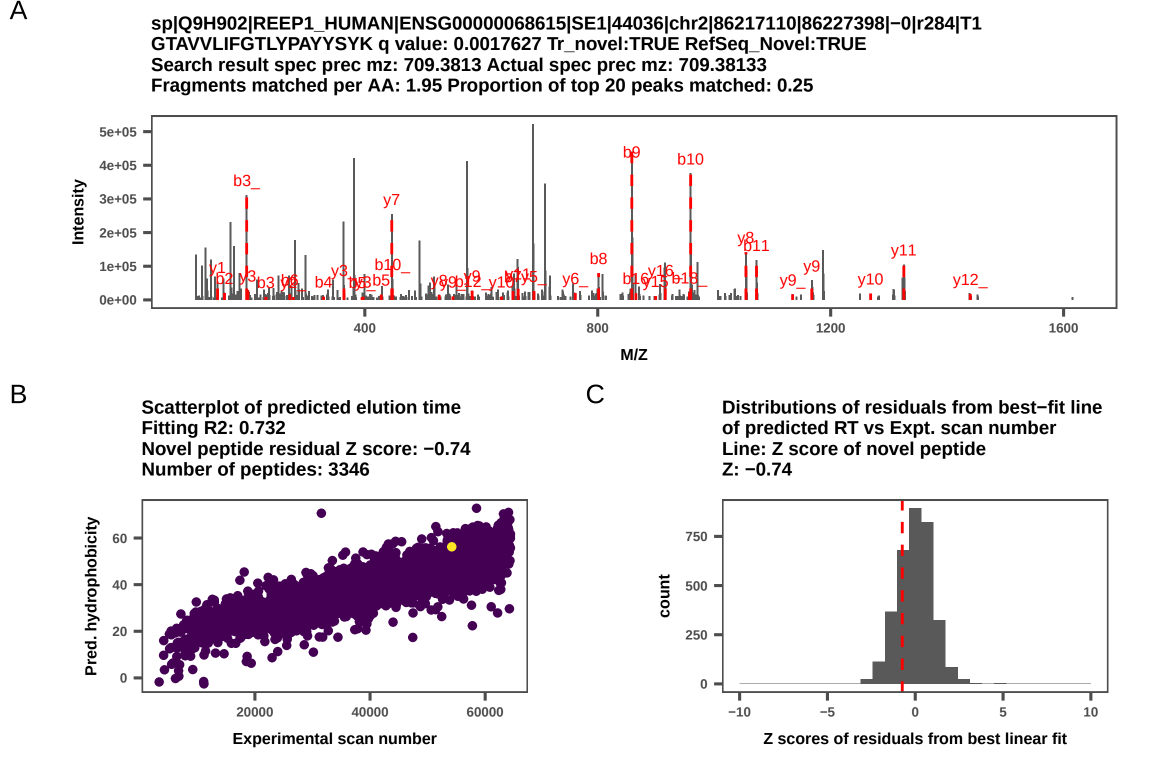
A
sp|Q9H902|REEP1_HUMAN|ENSG00000068615|SE1|44036|chr2|86217110|86227398|−0|r284|T1
GTAVVLIFGTLYPAYYSYK q value: 0.0017627 Tr_novel:TRUE RefSeq_Novel:TRUE
Search result spec prec mz: 709.3813 Actual spec prec mz: 709.38133
Fragments matched per AA: 1.95 Proportion of top 20 peaks matched: 0.25
Intensity
5e+05
4e+05
3e+05
2e+05
1e+05
0e+00
400
800
1200
1600
M/Z
b3_
y7
b9
b10
b8
y8
b11
y11
y10
y12_
B
Scatterplot of predicted elution time
Fitting R2: 0.732
Novel peptide residual Z score: −0.74
Number of peptides: 3346
Pred. hydrophobicity
Experimental scan number
20000
40000
60000
C
Distributions of residuals from best−fit line
of predicted RT vs Expt. scan number
Line: Z score of novel peptide
Z: −0.74
count
750
500
250
0
−10
−5
0
5
10
Z scores of residuals from best linear fit
